# Anomalous Origin of the Right Coronary Artery From the Mid-Portion of the Left Anterior Descending Artery

**DOI:** 10.7759/cureus.8794

**Published:** 2020-06-24

**Authors:** Rozi Khan, Nauman Siddiqi, John Wang

**Affiliations:** 1 Internal Medicine, MedStar Union Memorial Hospital, Baltimore, USA; 2 Internal Medicine, Bolan University of Medical and Health Sciences, Quetta, PAK; 3 Department of Cardiology, MedStar Union Memorial Hospital, Baltimore, USA

**Keywords:** "left anterior descending artery origion from rca", "anomalous coronary artery"

## Abstract

Coronary artery anomalies (CAAs) are rare findings and usually diagnosed incidentally on coronary angiograms for other cardiac conditions in most cases. However, coronary anomalies are being increasingly reported with the invention of more advanced cardiac imaging techniques. The CT of the heart structure is the best modality to diagnose and track the exact course of the anomalous artery and to guide in proper management. Anomalous course of the right coronary artery (RCA) between the aorta and pulmonary artery may cause compression and require surgical intervention given the risk of myocardial ischemia and sudden death. In this report, we discuss the case of a 69-year-old female with no prior cardiac comorbidities. The patient had been referred from the primary care office for cardiac clearance to undergo bilateral knee replacement surgery. On further inquiry, she reported a history of murmur and stated that she had been having dyspnea on exertion over the last six months. Precordial examination revealed a 2/6 ejection systolic murmur. Transthoracic echocardiogram showed severe aortic stenosis. For further evaluation, she underwent a coronary angiogram, which showed right dominant coronary system, normal left main with no stenosis, a large septal branch that had anomalously originated from left main and coursing all the way to the apex, and the RCA originating from the mid-portion of the left descending artery. The cardiac CT scan showed the exact course of the anomalous origin of the RCA from the mid-left anterior descending artery (LAD). The RCA coursed anteriorly to the main pulmonary artery/right ventricular outflow tract to reach the right atrioventricular groove. The patient underwent transcatheter aortic valve replacement (TAVR) and was discharged in stable condition.

## Introduction

The incidence of coronary artery anomalies (CAAs) is a very rare finding, diagnosed incidentally in 1.3% of cases on angiography and cardiac CT [1,2]. Most of these anomalies have no clinical significance; however, they are associated with profound ischemia and sudden cardiac death in cases where the right coronary artery (RCA) travels between the pulmonary artery and aorta, causing mechanical compression [3]. Among these coronary anomalies, single coronary artery is the term used for coronary artery originating from a single coronary ostium of ascending aorta. The RCA originating from the left anterior descending (LAD) artery is an extremely rare coronary anomaly; in such cases, the RCA originates from the proximal or mid-portion of the LAD artery. So far only 40 cases have been reported in which the RCA originates from the LAD and only 15 cases where the RCA originates from the mid-portion of the LAD artery [4].

## Case presentation

Our patient was a 69-year-old female with no medical history of cardiac disease. She had been referred by her primary care physician for cardiac clearance to undergo knee replacement surgery. On further inquiry, she reported a history of murmur and having dyspnea on exertion over the last six months. Precordial examination revealed a 2/6 ejection systolic murmur.

She underwent a transthoracic echocardiogram that showed normal left ventricular function, mild-left ventricular hypertrophy (Video 1), and moderate to severe aortic stenosis with an aortic valve area 0.08 cm grade (Videos 2, 3). For further evaluation, she underwent a coronary angiogram, which showed right dominant coronary system and normal left main with no stenosis; significant findings included the RCA originating from the mid-portion of the left descending artery and a large septal branch that had anomalously originated from left main, coursing all the way to the apex (Videos 4, 5).

**Video 1:** Transthoracic echocardiogram - view 1 .

**Video 2:** Transthoracic echocardiogram - view 2

**Video 3:** Transthoracic echocardiogram - view 3

 

**Video 4:** Coronary angiogram - view 1

**Video 5:** Coronary angiogram - view 2

**Figure 1:**
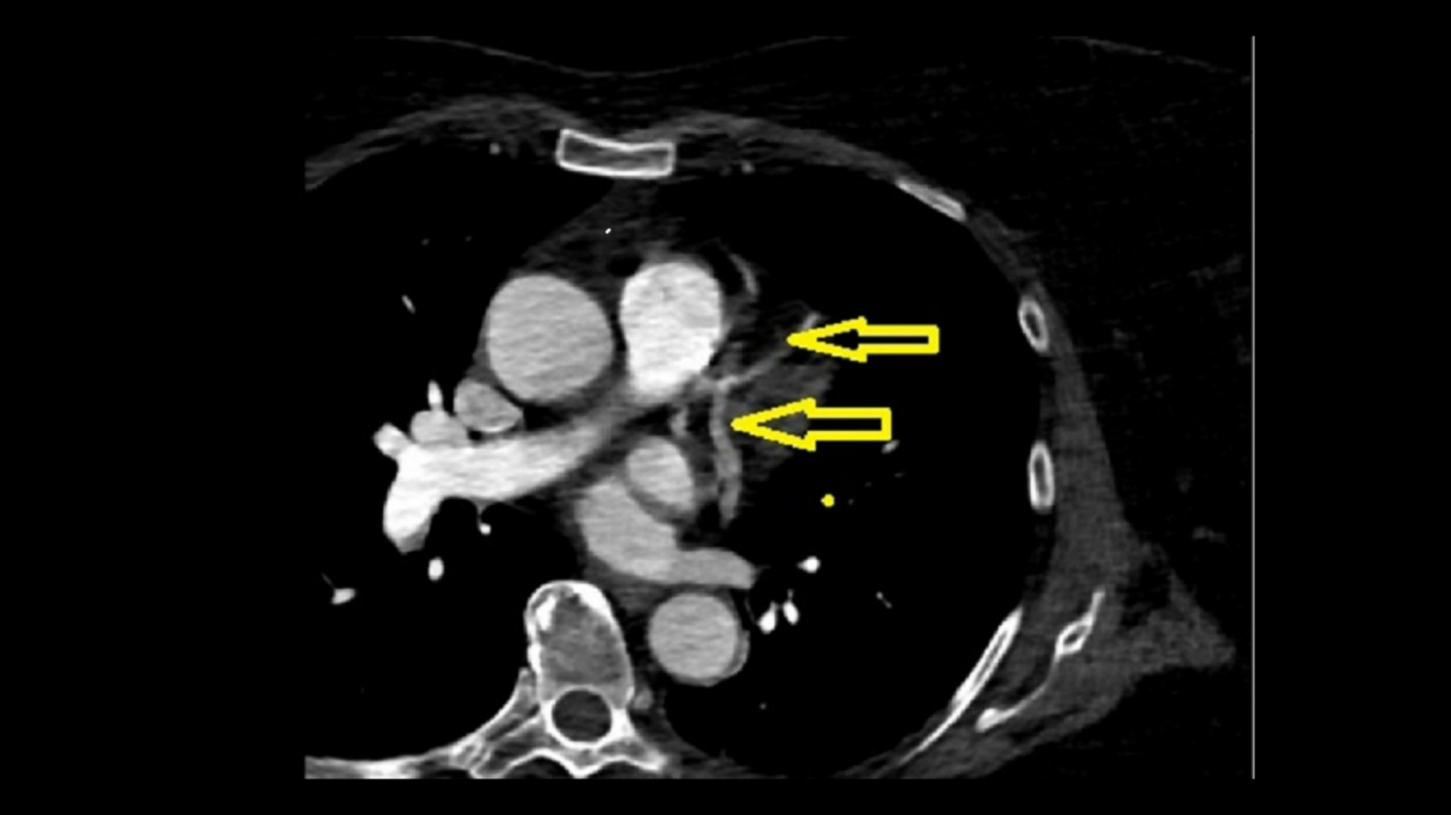
CT scan - view 1 The image shows the origin of RCA from mid-portion of LAD artery (top arrow points to LAD and bottom arrow points to RCA) CT: computed tomography; RCA: right coronary artery; LAD: left anterior descending

**Figure 2:**
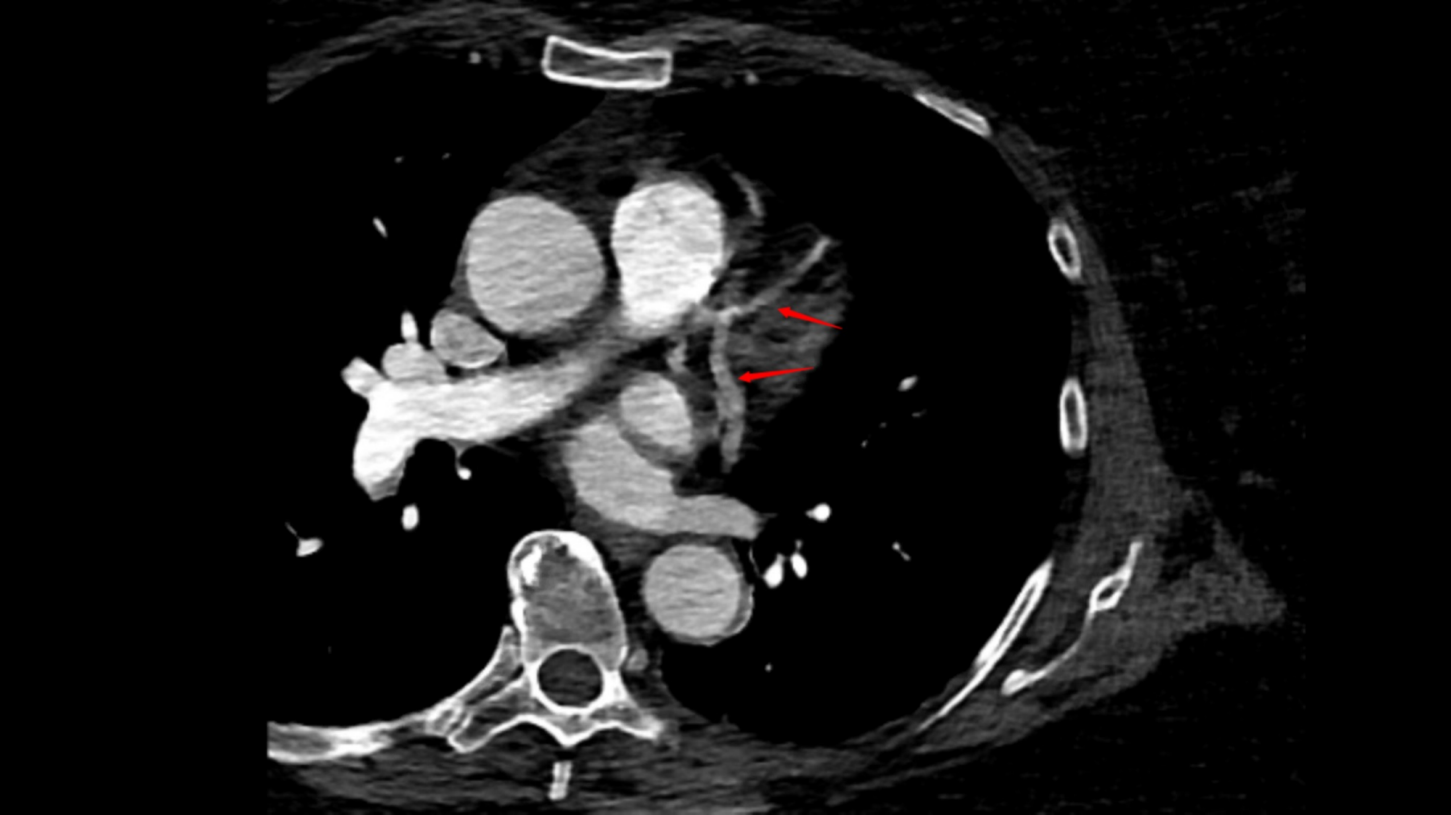
CT scan - view 2 Arrows in the image indicate the LAD artery and RCA. The point of origin of RCA from the LAD artery can be clearly seen CT: computed tomography; RCA: right coronary artery; LAD: left anterior descending

The patient subsequently underwent CT of the heart structure without contrast that showed heart without anomaly or defect, left ventricular hypertrophy, and anomalous origin of the RCA from the mid-LAD (Figures 1, 2). The RCA coursed anteriorly to the main pulmonary artery/right ventricular outflow tract to reach the right atrioventricular groove. The patient subsequently underwent transcatheter aortic valve replacement (TAVR) surgery and was discharged in stable condition.

## Discussion

CAAs are very rare among the general population. They are diagnosed in merely 0.16-1.3% of the patients referred for a coronary angiogram. Single coronary artery anomaly is even rarer with an incidence of 1.1-8.8% of all coronary artery anomalies [2], and only occurs in 0.024% of the general population [5]. The most common form of CAA involves the separate origin of the LAD and left circumflex (LCX) artery, with an incidence of 0.41%, followed by the LCX artery arising from the RCA, with an incidence of 0.37% [4]. Different classification systems have been developed for CAA but no single classification is widely adopted. The classification based on anatomical features divides the CAA into (1) anomalies of the ostium, (2) anomalies based on coronary artery origin from the aorta, and pulmonary artery, (3) duplication of the RCA or LAD or LCX, and (4) congenital absence or hypoplasia [6].

Generally, the anomalous RCA originates from proximal to mid-LAD as in our patient. Significantly, 97% of the patients with the RCA originating from LAD has a structurally normal heart although single coronary artery is associated with other congenital anomalies such as bicuspid valve, Tetralogy of Fallot, and transposition of great arteries [7]. In most cases, the RCA originating from LAD is asymptomatic, diagnosed incidentally, and has a better prognosis except if the RCA is passing between the aorta and pulmonary artery. This course of the RCA anomaly is malignant and prone to compression and at higher risk to develop atherosclerosis, myocardial ischemia, and sudden cardiac death even without underlying atherosclerosis [7,8]. CAA is considered to be the second most common cause of death in young athletes [9]. It is hypothesized that coronary anomaly-associated myocardial ischemia and sudden cardiac death is secondary to different pathological changes in an anomalous coronary artery, e.g, coronary vasospasm, slit-like orifice, and the acute angle of origin of anomalous artery form parental artery [7]. The latter two are considered to be the main culprits behind sudden cardiac death [10].

As previously discussed, most of the RCA anomalies are diagnosed incidentally on coronary angiogram. Once diagnosed, it is very important for the physician to perform a CT scan of the cardiac structure to have a better idea of the full course of the anomalous coronary artery and its anatomic relationship with great vessels. CT cardiac structure is the gold-standard investigational tool for the diagnosis of coronary anomalies [8], and recent guidelines recommend the use of CT angiogram (CTA) for a detailed evaluation of coronary anomalies [3].

Most of the RCA anomalies are asymptomatic and hardly need treatment. However, in cases of associated cardiac ischemia secondary to the anomalous artery, medical management and percutaneous intervention are needed. Treatment of the malignant type where the anomalous RCA is passing between pulmonary artery and aorta involves surgical management [5]; fortunately, no such cases have been reported so far [2]. Based on an extensive search of the literature, we have found that the anomalous origin of the RCA from the LAD artery with a separate small anomalous branch arising from the RCA has not been demonstrated in any of the previous case reports.

## Conclusions

CAA is a very rare form of congenital heart anomaly. Furthermore, the anomalous origin of the RCA from the LAD artery is a rare form of CAA in which the anomalous RCA arises from the proximal or mid-portion of the LAD. This anomaly often has a benign course except when RCA traveling between the pulmonary artery and aorta involves surgical management. Two different theories have been formulated about the risk of atherosclerosis in coronary anomalies, one in favor and one against; but it is generally thought that the acute take-off angle of CAA origin could constitute the risk factor for atherosclerosis.
